# Comparative analysis of viral infection outcomes in human seminal fluid from prior viral epidemics and Sars-CoV-2 may offer trends for viral sexual transmissibility and long-term reproductive health implications

**DOI:** 10.1186/s12978-021-01172-1

**Published:** 2021-06-10

**Authors:** James Frederick W. Pike, Emily L. Polley, David Y. Pritchett, Arnav Lal, Blake A. Wynia, William E. Roudebush, Renee J. Chosed

**Affiliations:** 1 Department of Biomedical Sciences, University of South Carolina School of Medicine Greenville, 607 Grove Rd., Greenville, SC 29605 USA; 2 School of Arts and Sciences, University of Pennsylvania, Philadelphia, PA 19104 USA; 3 Department of Surgery, Division of Urology, PRISMA Health Upstate, 701 Grove Rd., Greenville, SC 29605 USA

**Keywords:** COVID-19, Coronavirus, SARS-CoV-2, Seminal fluid, Zika, Ebola, SARS, Fertility

## Abstract

**Background:**

Viral detection in seminal fluid indicates their potential for both sexual transmission and impairment of reproductive health. Review of the mechanistic entry, sexual transmission and viral impacts for patients during major recent viral outbreaks of Zika virus (ZIKV), Ebola virus (EBOV), severe acute respiratory syndrome (SARS)-coronavirus (CoV), and SARS-coronavirus 2 (CoV-2) (the virus which causes COVID-19) provides a framework to discuss this potential.

**Aim:**

Comparative analysis of prior viral presence on seminal fluid against current (preliminary) findings for SARS-CoV-2 to predict biological implications of the novel coronavirus upon current sexual transmissibility, viral presence, and reproductive health.

**Methodology and findings:**

Literature review was conducted using PubMed and Google Scholar databases. ZIKV and EBOV were found to be present in semen and to be sexually transmitted, leading the World Health Organization (WHO) to update their guidelines on prevention of the two viruses to include refraining from sexual contact. There are conflicting studies regarding the presence of SARS-CoV in male reproductive tissue, but it has been linked to testicular atrophy and orchitis. To date, two studies have detected SARS-CoV-2 RNA in semen, while seven studies have reported no positive detection.

**Conclusions:**

Though unlikely in the majority of cases, SARS-CoV-2 can potentially be present in seminal fluid, although there are no reports of sexual transmission to date. Prior epidemics raise significant concerns regarding the long-term reproductive health capacity for patients who are affected by entry of Sars-CoV-2 into the reproductive tract, therefore more study is needed to clarify the impacts to reproductive health.

## Plain Language Summary

This review describes the detection of viruses in seminal fluid and their sexual transmission, focusing on the major viral outbreaks of Zika virus (ZIKV), Ebola virus (EBOV), severe acute respiratory syndrome (SARS)-coronavirus (CoV), and SARS-coronavirus 2 (CoV-2). ZIKV and EBOV were found to be present in semen and to be sexually transmitted, leading the World Health Organization (WHO) to update their guidelines on prevention of the two viruses to include refraining from sexual contact. There are conflicting studies regarding the presence of SARS-CoV in male reproductive tissue, but it has been linked to testicular atrophy and orchitis. To date, two studies have detected SARS-CoV-2 RNA in semen, while seven studies have reported no positive detection. More studies must be completed to accurately determine its risk of sexual transmission to ensure mitigation of further transmission and understand the long-term implications of SARS-CoV-2 on the reproductive health of recovered patients.

## Background

There has been reported evidence of 27 different viral species present in human semen [1]. Epidemiologically, it is important for the public to know whether a virus can be transmitted sexually, especially if it can be transmitted while the patient is an asymptomatic carrier, assumed to be fully recovered, or interacting with others during the infection’s incubation period. Specific areas of the body, such as the testis, eyes, CNS, and placenta, have immune privilege [2], hypothesized as an evolutionary adaptation which decreases the immune response in these organs and tissues in order to preserve their function and limit autoimmunity. Therefore, this decreased immune response implies that viruses can persist in the male reproductive tract, and thus a patient may have a prolonged ability to transmit the virus sexually [2]. The novel coronavirus pandemic has provoked research regarding transmission, and it is important for the public to know whether sexual transmission is possible. In this review, we compare the viral presence and implications of ZIKV, EBOV, SARS-CoV, and SARS-CoV-2, in human seminal fluid and discuss potential implications for overall reproductive health.

## Methods

A review of the literature involving ZIKV, EBOV, SARS-CoV, and SARS-CoV-2 was conducted. Each virus was searched separately alongside keywords relating to the content of this manuscript (e.g., testis, semen, seminal fluid, sexual transmission). Search databases included PubMed and Google Scholar.

Inclusion criteria for this search were: (1) original manuscripts involving these emerging viruses which addressed their effects on the male reproductive tract and/or potential for sexual transmission; (2) studies published between January 1, 1996-August 1, 2020 to encompass available literature from multiple EBOV outbreaks up to the completion of this manuscript; (3) studies in English. Primary exclusion criteria were: (1) studies published prior to 1996; (2) abstract-only manuscripts and review articles; (3) studies published in non-English languages.

### Zika virus (ZIKV)

The first reported case of sexual transmission of ZIKV was published in 2011 [3]. Upon returning to the United States from Senegal, a man reported having vaginal intercourse with his wife. Soon after, both patients began showing symptoms and tested positive for ZIKV. As competent mosquito vectors for ZIKV are not native to the geographic region of residence of the couple, transmission of the virus was presumed to be sexual [3]. Following this report, the 2014 outbreak in the Americas lead to an influx of case reports confirming not only that ZIKV was sexually transmitted, but that male-to-female transmission was the most common form of sexual transmission [4]. From 2014 to 2017, the United States had reached 52 cases of confirmed sexual transmission of ZIKV, prompting research investigating the presence of ZIKV in both the immune-privileged testes and seminal fluid [5].

The immune privilege of the male testes raised concerns that they may harbor persistent ZIKV reservoirs offering an opportunity for viral sexual transmission over extended periods. Recent studies have shown the TAM (TYRO3, AXL, and MERTK)-family receptor tyrosine kinase AXL to be the primary ZIKV entry cofactor [6]. AXL is highly expressed in Sertoli cells (SCs), a major component of the blood-testis barrier (BTB) which provides protection from the entry of pathogens into the seminiferous tubules. Siemann et al. have demonstrated, in vitro*,* that SCs can be infected by ZIKV and maintain high viral titers up to day 9 after infection. Additionally, utilization of an established in vitro BTB model demonstrated that cell-free ZIKV is efficiently released on the adluminal side of infected SCs [7]. Furthermore, studies claim that ZIKV is not only present in the semen of infected patients but remains in the male reproductive tract for a considerable amount of time after symptoms have subsided. In a longitudinal 6-month study examining ZIKV load and immunologic profile in the blood, plasma, and semen of one man, Mansuy et al. recorded more prolonged (168 days) and substantial (1.04 × 10^5^ copies/mL) amounts of viral RNA shedding in the semen than in the blood (100 days, 9.4 × 10^3^ copies/mL) of the patient [8]. On a larger scale, Mead et al. analyzed semen samples from 184 men with symptomatic ZIKV infection. ZIKV RNA was found in the semen of 60 (33%) subjects, with most samples being provided between 30 and 100 days. However, when specifying to men who provided samples within 30 days of illness onset, ZIKV RNA detection increased to 22 out of 36 men (61%). ZIKV RNA in samples submitted after 90 days of illness onset decreased substantially to 9/132 (7%), but was nevertheless notably present in some individuals despite significant time after illness. RNA was even found in the sample from one study participant after 281 days [9]. Currently the WHO recommends practicing safer sex or abstinence for those who are returning from areas of active ZIKV transmission; a period of six months (~ 180 days) for men and two months (~ 60 days) for women [10]. While in the vast majority of cases this is most likely an effective precaution, more research is needed to fully understand sexual transmutability of ZIKV.

### Ebola virus (EBOV)

First isolated in the 1970s, EBOV continues to cause outbreaks, the majority of which have occurred in several regions of Africa. Most notable and most severe was the 2014–2016 outbreak of EBOV in West Africa, which claimed the lives of over 11,000 people. Since that time, the body of research on the pathogenesis and epidemiology of EBOV has expanded. EBOV is known to enter host cells by glycoprotein fusion with specific cell membranes and subsequent endosome formation [11]. The detection method most commonly used in studies that evaluate the persistence of EBOV is reverse transcription polymerase chain reaction (RT-PCR), which is capable of detecting viral RNA unique to EBOV. Because the testes are immune-privileged, EBOV has been found to persist in testicular tissues long after clinical convalescence has occurred. The exact mechanism of viral tropism has not yet been found, but it has been hypothesized that persistence of EBOV is established in the interstitium of the male reproductive tract (seminal vesicle, epididymis, prostate gland, testis) and is shuttled to the seminal fluid via infected tissue macrophages [12]. One study by Rodriguez et al. tracked patients after the 1995 EBOV outbreak in the Democratic Republic of the Congo and found convalescent patients to have EBOV RNA via RT-PCR present in seminal fluid samples as late as 101 days after onset of disease [13]. Another study conducted in a similar timeframe of this outbreak by Rowe et al. found evidence of EBOV RNA via in seminal fluid up to 91 days after disease onset [14]. Sample sizes in both of these studies were limited, with analysis of semen samples of 12 patients in Rodriguez et al. and five patients in Rowe et al.

More recently, several studies have found that EBOV RNA may persist longer in the seminal fluid than previously thought. The number of patients with RNA detected by RT-PCR decreases over time after convalescence, but viral RNA can still persist for up to 16–18 months [15]. Soka et al. observed one convalescent EBOV patient to have a positive RT-PCR semen sample at 565 days after discharge from an EBOV treatment unit [16]. Interestingly, Keita et al. found a positive and significant relationship between older age and the length of the period of viral RNA detection in semen. The mechanism of this association has not been elucidated. According to their study, almost all male survivors of EBOV can be considered to be RNA-positive for up to 3 months, and up to 70% can be considered to be positive for up to 6 months post-discharge [17].

Most studies evaluating the persistence of EBOV RNA in semen cannot confirm the presence of viral particles and thus are incapable of directly correlating a positive result with infectivity. Cell culture techniques can be used to evaluate the seminal fluid for presence of virus particles which are known to be infectious. Kainulainen et al. described a novel reporter cell line used to identify EBOV particles in bodily fluid samples, and this could be potentially useful in providing better surveillance of EBOV spread (should another EBOV outbreak occur), though the clinical use of this method has not yet been described in the literature [18]. Additionally, in 2017, Sissoko et al. executed a study where semen from convalescent men was injected into the peritoneal cavities of severe combined immunodeficiency (SCID) knockout mice, as only intact viral particles (as opposed to viral RNA) would be able to cause a clinical EBOV infection [19]. Their experiments in mice indicated shedding of infectious viral particles in the seminal fluid for up to 200 days after disease onset. This suggests active replication and thus viral persistence in the male reproductive tract, though it does not directly test for viral particles in convalescent patient semen [19]. However, this study supports the theory that sexual transmission of EBOV is more than merely theoretical. True sexual transmission of EBOV has been documented once in the literature [20]. In 2015, a 44-year-old woman from Sierra Leone became infected with EBOV after an unprotected sexual encounter with a male EBOV survivor [20, 21]. Viral genomes sequenced from the patient and the survivor were consistent with a pattern of direct transmission, and the patient’s history was otherwise negative for encounters with diseased or convalescent individuals. It was therefore inferred that infectious EBOV particles were present in the survivor’s semen at least 179 days after onset of disease [20, 21]. From these studies, it can be concluded that surveillance of seminal fluid of male EBOV survivors could be useful in mitigating the next outbreak via more rigorous contact tracing and public communication measures. The WHO updated their guidelines for prevention of EBOV transmission in 2016 to include practicing safe sex and hygiene for 12 months from onset of symptoms or until two negative semen tests for EBOV were reported [22]. Comprehensive, appropriate patient education upon discharge from EBOV treatment units regarding safe sexual practices could help to limit the number of EBOV cases in the future.

### SARS-CoV

Before the detection and onset of the SARS-CoV-2 pandemic, not much was known about novel coronaviruses with regard to their effects on the testes and potential for sexual transmission. Data with regard to the effects of SARS-CoV on the testes specifically is limited. In 2004, Ding et al. found SARS-CoV to be present in many different organs and tissues of deceased patients including lung, trachea, bronchus, stomach, kidney, small intestine, pancreas, adrenals, and liver, but to be absent in pathologic specimens of testes [23]. This finding was puzzling to the researchers since SARS-CoV enters host cells through the angiotensin converting enzyme 2 (ACE2) receptor. ACE2 is abundantly expressed in the organs and tissues where SARS-CoV RNA was detected in the study by Ding et al. including the lungs, gastrointestinal (GI) tract, and kidney, but it is also at high levels in testicular tissue [24]. While Gu et al. also noted the absence of SARS-CoV viral particles in testicular specimens of deceased patients, focal testicular atrophy was noted to be present in the specimens of all seven male patients positive for SARS [25]. In 2006, Xu et al. documented six cases of orchitis in males who had succumbed to SARS. Testes analyzed from these SARS patients displayed marked germ cell destruction with diminished spermatozoon presence, thickened basement membranes, and leukocytic infiltration [26]. Due to these study results and the lack of studies regarding sexual transmission of SARS-CoV, the possible transmission of SARS-CoV through viral presence in the reproductive tract cannot be confirmed.

### SARS-CoV-2

The worldwide outbreak of SARS-CoV-2 has led to a need for research regarding its properties and mechanisms of transmission in order to establish treatments and prevention guidelines. One of the main mechanisms used by the SARS-CoV-2 virus to enter cells is through the ACE2 receptor [27, 28]. SARS-CoV-2 utilizes a spike protein S1, similar to SARS-CoV, in order to bind to the ACE2 receptor, however it does so with 10–20 times the binding affinity of SARS-CoV [28]. The ACE2 receptor has been shown to be highly expressed in renal tubular cells, Leydig cells, and seminiferous tubules, proposing the possible vulnerability of the male reproductive system to SARS-CoV-2 [29]. Importantly, the testes are a site of immune privilege, leading to viral protection from the inflammatory immune response and therefore an extended presence in the tissue.

There have been nine total studies published regarding the presence of SARS-CoV-2 in semen. A study published in May 2020 in JAMA by Li et al. using RT-PCR detected SARS-CoV-2 in the semen of 6 out of 38 men, four at the acute stage and two in the recovery stage of SARS-CoV-2 [30]. This study showed that it is possible for SARS-CoV-2 to enter semen, and therefore establish sexual transmission. It is important to note, however, that these patients were hospitalized with severe disease, and the two patients who had SARS-CoV-2 detected in their semen after recovery had their samples collected 2–3 days after recovery, a relatively short time interval from acute infection [30]. The remaining studies, mostly reporting patients with mild disease, have reported no evidence of SARS-CoV-2 in semen, until the most recent study published in February of 2021 had positive detection of the virus in 1/15 patients [31–38]. Pan et al. reported a sample size of 34 with mild disease. Sample collection time was 8–75 days after positive nasal swab test, median 31 days, and all 34 patients tested negative for SARS-CoV-2 RNA in semen [31]. Holtmann et al. studied 18 patients positive for SARS-CoV-2 nasal swab, 14 with mild disease and 4 with moderate disease. All samples were collected 8–54 days after absence of symptoms, and there were no positive results for SARS-CoV-2 RNA in the semen [32]. A third study by Song et al. included 12 semen samples, 11 from men with mild/common disease and 1 from an asymptomatic man. All 12 patients had positive anti-2019-nCoV IgG but negative anti-2019-nCoV IgM, indicating the virus had largely been cleared and was not in the acute phase. One additional patient was also included who had died during SARS-CoV-2 infection and he had tested positive for both IgM and IgG in serum, and samples of his testis were taken. His samples, along with the semen of the 12 other patients, showed no detectable 2019-nCoV RNA [33]. Guo et al. reported a study consisting of 23 patients. 18 were diagnosed with mild disease and 5 with moderate, but none had severe or critical disease. In 11 patients, viral detection had cleared from sputum and fecal specimens upon semen sample collection, and 12 still tested positive for the virus in sputum and fecal specimens. The median interval from diagnosis to providing semen samples was 32 days, and all 23 patients tested negative for SARS-CoV-2 RNA in semen specimens [34]. Rawlings et al. enrolled 6 participants in a study seeking to detect SARS-CoV-2 RNA in both semen and saliva samples. All of the participants were outpatients at the time of sample collection. They concluded that all 6 tested positive for SARS-CoV-2 RNA in oral secretions 6–17 days after symptom onset, but there was no detection in semen of any of the patients [35]. In a study by Ma et al., semen specimens were collected from 12 patients. One of the 12 had mild disease, while the remaining 11 had moderate. The time between symptom onset and semen collection ranged from 56 to 109 days, and for all 12 patients, no SARS-CoV-2 RNA was detected in their semen [36]. Kayaaslan et al. reported data from 16 patients with semen samples obtained within 7 days of positive nasopharyngeal test, with a median of 1 day. Eleven patients had mild disease and 5 had moderate, and all 16 samples tested negative for SARS-CoV-2 RNA [37]. The latest study by Machado et al. is the second after the Li study to detect the presence of SARS-Cov-2 viral RNA in human semen. They reported detection of SARS-CoV-2 RNA in 1/15 patients with tests taken 0–14 days from symptom onset. Two patients were asymptomatic and the other 13 had either mild or moderate disease. They did not report which specific patient tested positive for SARS-CoV-2 RNA in the semen [38]. Results of SARS-CoV-2 studies are summarized in Table 1.

**Table 1:** Current published data regarding SARS-CoV-2 detection in semen samples and the parameters used in the studies

	Li et al. 2020 (JAMA)	Pan et al. 2020 (F + S)	Holtmann et al. 2020 (F + S)	Song et al. 2020 (Biol Reprod)	Guo et al. 2020 (Andrology)	Kayaaslan et al. 2020 (Urol Int)	Rawlings et al. 2020 (OFID)	Ma et al. 2021 (J Med Virol)	Machado et al. 2021 (Infect Dis Rep)
Location	Shangqiu, China	Wuhan, China	Duesseldorf, Germany	Wuhan, China	Wuhan, China	Ankara, Turkey	California, USA	Wuhan, China	Arkansas, USA
Sample Size	38	34	34 (18 with disease)	13 (12 semen samples)	23	16	6	12	15
Disease Type	Severe: 38 Positive Tests: Acute Stage: 4 Recovery Stage: 2	Mild: 34	Mild: 14 Moderate: 4	Asymptomatic: 1 Mild: 11	Mild: 18 Moderate: 5	Mild: 11 Moderate: 5	Unknown	Mild: 1 Moderate: 11	Asymptomatic: 2 Mild to Moderate: 13
Method of Disease Confirmation	RT-PCR	RT-PCR	RT-PCR	RT-PCR	RT-PCR	RT-PCR	RT-PCR	RT-PCR	RT-PCR
Detection in Semen?	6/38	0/34	0/18	0/12 0/1 In Testis Sample	0/23	0/16	0/6	0/12	1/15
Days after Diagnosis	6–16	8–75	8–54	Unknown	27–33	0–7	6–17	56–109	0–14

Evidence is also emerging that SARS-CoV-2 may cause direct damage to testicular tissues, which could have negative implications for long-term fertility in recovered patients. In a fashion similar to SARS-CoV, pathological specimens of testes collected from patients who died from SARS-CoV-2 infection revealed evidence of testicular injury [39, 40]. Upon examining postmortem testicular specimens of SARS-CoV-2 patients, Nie et al. found proteomic evidence of downregulation of five key genes essential for proper testosterone production and spermatogenesis to occur [39]. Ma et al. examined testicular tissue of 5 deceased SARS-CoV-2 patients and compared the specimens to those of age-matched controls [40]. In the SARS-CoV-2 patients, they found marked germ cell loss and sloughing into the lumen of the seminiferous tubule and significantly higher numbers of apoptotic cells, compared to normal germ cell morphology and architecture in the controls, but interestingly the Sertoli cell population was spared [40]. More research is needed to reconcile this finding with the proposed notion that SARS-CoV-2 infection causes compromise of the BTB. They also used IHC staining for the S1 spike protein of SARS-CoV-2, and the testicular sections from SARS-CoV-2 patients stained positively, indicating direct infection of testicular cells by SARS-CoV-2 [40]. However, more research is needed because it is still unclear whether these deleterious effects on the testis are due completely to direct infiltration of the virus itself, local immune response to the virus, severe systemic illness (e.g., fever, profound multisystem inflammatory response), or a combination of these.

Of note, it is possible that the prostate also plays a role in the sexual transmissibility implications of SARS-CoV-2 infection. The prostate is known to express the transmembrane protease, serine 2 (TMPRSS2), which is androgen-sensitive and is expressed at high levels in the prostate [41]. SARS-CoV-2 is known to enter cells through the ACE2 receptor and TMPRSS2 for S protein priming, as demonstrated by Hoffmann et al. [42]. Upon surveying prostatic cellular expression of both ACE2 and TMPRSS2, Song et al. found a population of these “double-positive” cells in prostatic epithelium, suggesting that these cells could serve as a potential reservoir for SARS-CoV-2 infiltration, damage, and transmission through prostatic secretions [43]. However, these “double-positive” cells only constitute about 0.07% of all prostate epithelial cells, so more research is needed to determine the clinical significance of this finding [43].

## Conclusion

The worldwide spread of SARS-CoV-2 from Wuhan, China in December 2019 to April 2021 led to over 130 million confirmed cases and nearly 3 million confirmed deaths worldwide [44]. It is imperative to understand the means of transmission of the virus in order to properly inform the public on appropriate safety measures. One of the most important epidemiological elements to preventing the spread of a disease involves the determination of methodology of disease transmission. Sexual transmission may play a significant role in the spread of an infectious disease, and this may be further exacerbated by the immune privilege of the male reproductive tract. Prior viral infections such as ZIKV and EBOV have demonstrated the capacity to spread via sexual contact for long periods of time after symptom onset. Several studies have also elucidated some of the likely mechanisms involving the entry of these viruses into the male reproductive tract including the TAM/AXL pathway for Zika Virus and glycoprotein fusion with cell membranes with endosome formation for EBOV. Moreover, the information that viral load or viral RNA was present within the reproductive tract along with detection of sexual transmission led to clarification from global health entities regarding updating safety policies to reduce the spread of these viruses.

There have been 9 studies published regarding the presence of SARS-CoV-2 in the semen. 7 have reported semen samples negative for SARS-CoV-2, while 2 did detect the presence of SARS-CoV-2 RNA in semen [30–38]. All of the patients in the studies that reported no detection of SARS-CoV-2 in their semen had what were considered mild or moderate symptoms. No patients were included with severe or critical disease. Prior research has associated high viral loads with more severe disease symptoms, postulating that a certain viral threshold may significantly increase the likelihood for the virus to cross the BTB [45]. In the Li study, patients were hospitalized with severe disease, and all semen samples from recovering patients that were found positive for SARS-CoV-2 viral RNA were collected 2–3 days after recovery. In August of 2020, Kayaaslan et al. sought to answer the question of whether the length of time since infection affected transmission from the body into semen [38]. They theorized that since the patients in the Li study with reported RNA detection in semen were either at the acute phase of infection or within three days of release from the hospital, viral load in patients would be higher in the acute phase and therefore the virus would have a greater chance of entering the semen. The Kayaaslan study reported no detection in semen within 7 days of positive nasopharyngeal test, providing more evidence that patients with present or past SARS-CoV-2 may not be at risk for sexual transmission. However, like the other 7 studies that had negative detection of RNA in semen, they had no patients with severe disease and only 5/16 with moderate. In the most recent Machado study in February of 2021, however, all samples were collected within 2 weeks of symptom onset from patients with mild to moderate symptoms and they revealed positive detection of SARS-CoV-2 RNA in 1/15 samples [38]. While this study was limited in size, it is important to note there have been no reports of sexual transmission of SARS-CoV-2 to date.

Additionally, previous studies on SARS-CoV have shown its detrimental effect on the male reproductive tract, and reports are beginning to show degradation within the male reproductive tract due to SARS-CoV-2 as well [39–43]. These findings have two implications. First, from an epidemiological perspective, the significant injury observed in postmortem SARS-CoV-2 patients seems to indicate the possible presence of SARS-CoV-2 within the male reproductive tract, at least for some patients. Second, the deterioration of the male reproductive tract in postmortem SARS-CoV-2 patients may imply that if the virus does cross the BTB, then damage may be incurred upon the future reproductive health of the individual.

Because many patients with SARS-CoV-2 can either recover or be asymptomatic and never require treatment, it is important to continue to investigate the health effects of SARS-CoV-2 upon the reproductive tract in light of the preliminary analyses conducted. These studies provide potential that the BTB is not restrictive to SARS-CoV-2, and it is important to determine the spread and rate of transmission. It is possible that, in a similar fashion to ZIKV and EBOV, the virus may persist for an extended period of time in the male reproductive tract due to immune privilege [2]. Additionally, while the reproductive potential of most SARS-CoV-2-recovered patients may not have been significantly affected, some recovered patients may later face difficulty with infertility and their overall reproductive health. A longitudinal analysis of viral load and semen parameters may provide more compelling evidence towards the possibility of sexual transmission. It is also important to study sexual transmission of SARS-CoV-2, as the presence of a virus in semen may not automatically lead to sexual transmission from person to person [46]. Continued research into the long-term reproductive health of SARS-CoV-2-recovered patients appears warranted from the preliminary data. Further studies on SARS-CoV-2 transmission through sexual activity could possibly lead to public health policy revisions, with the ultimate goal of providing the most effective prevention guidelines to the public.

## Data Availability

Not applicable.

## Abbreviations

ZIKV: Zika virus
EBOV: Ebola virus
SARS: Severe acute respiratory syndrome
CoV: Coronavirus
CoV-2: SARS-coronavirus
SARS-CoV-2: The virus which causes COVID-19
WHO: World Health Organization
CNS: Central nervous system
RNA: Ribonucleic acid
TAM: TYRO3, AXL, and MERTK-family receptor tyrosine kinase AXL
SC: Sertoli cells
BTB: Blood-testis barrier
RT-PCR: Reverse transcription polymerase chain reaction
IHC: Immunohistochemistry
